# Obituary

**Published:** 1910-01-29

**Authors:** 


### Obituary.

The death is announced of Dr. William Page-May, of 14 Great Cumberland Place. Dr. May gained a University Scholarship and a gold medal in medicine in 1888, and in 1890 was a gold medallist again when he took the degree of M.D. at London. He was a Fellow of University College, London, among other distinctions, and had been Registrar and Pathologist at the City of London Hospital for Diseases of the Chest, and house physician to the National Hospital for the Paralysed. Apart from several monographs he was also author of "Helouan and the Egyptian Desert."

The death is announced of Mr. Stanley Bean Atkinson at the age of thirty-six. Mr. Atkinson was an M.B. and B.Sc. of London University, and an M.A. and LL.M. of Cambridge. He gained a junior entrance scholarship at St. Bartholomew's Hospital in 1892 and a junior scholarship in anatomy and physiology in the following year, and took his diploma in 1902. He was also called to the Bar in 1899. He represented the Mile End Board of Guardians on the Metropolitan Asylums Board, and was for a time senior house physician to the City of London Hospital for Diseases of the Chest. He was a member of a number of learned societies, and was the author of "Golden Rules of Medical Evidence," published in 1906, "The Law in General Practice," 1908, etc.

